# The burden and characteristics of HIV-infected COVID-19 patients at a tertiary care hospital in sub-Saharan Africa—A retrospective cohort study

**DOI:** 10.1371/journal.pone.0273859

**Published:** 2022-08-31

**Authors:** Alok Iyer, Jasmit Shah, Reena Shah

**Affiliations:** 1 Department of Internal Medicine, Aga Khan University, Nairobi, Kenya; 2 Brain and Mind Institute, Aga Khan University, Nairobi, Kenya; University of Cape Town Faculty of Science, SOUTH AFRICA

## Abstract

**Background:**

After the first case of COVID-19 caused by the novel SARS-CoV-2 virus was discovered in Wuhan, China, in December 2019, the disease spread viciously throughout the world. Little is known about the impact of HIV infection on the clinical outcomes of patients co-infected with SARS-CoV-2. Studying the characteristics and outcomes of COVID-19 among HIV-positive patients is key to characterising the risk of morbidity and mortality of HIV-positive patients from COVID-19.

**Methods:**

In this retrospective cohort study, we included patients admitted to Aga Khan University Hospital, Nairobi, with laboratory-confirmed COVID-19 infection and who had consented to HIV screening. We compared the prevalence and characteristics of HIV patients with those of non-HIV patients and described the results for both groups.

**Results:**

In our sample of 582 patients, the mean age was 49.2 years (SD = 15.2), with 68% of the sample being men. The cumulative HIV prevalence was 3.7%, and the most common symptoms were cough (58.1%), fever (45.2%), difficulty in breathing (36.8%) and general body malaise (23.9%). The most common comorbidities included hypertension (28.5%), diabetes mellitus (26.1%), and heart disease (4.1%). Most participants (228 or 49.5%) had mild COVID-19, and the mortality rate was 5%. Overall, there were no statistically significant differences in demographic characteristics, clinical characteristics, and outcomes between HIV-positive and HIV-negative patients.

**Conclusions:**

There was a 3.7% prevalence of HIV in COVID-19 positive patients. Demographic characteristics and clinical outcomes were similar between the two groups. Future studies should seek to achieve larger samples, include multiple study sites and conduct subgroup analyses based on the immunologic status of HIV-positive patients.

## Introduction

The World Health Organization (WHO) declared COVID-19 a pandemic in March 2020, with cases worldwide rising at an alarming rate (over 526 million confirmed cases) and claiming over six million lives as of May 2022 [1]. Kenya has had about 330,000 cases reported and about 5600 deaths as of May 2022 [2].

In a retrospective study from Wuhan (China) involving 210 patients with COVID-19, Wu et al. compared risk factors related to acute respiratory distress syndrome (ARDS) development and ARDS-associated death to those who did not develop ARDS [3]. They found that compared to patients without ARDS, patients with ARDS had a higher proportion of comorbidities, including hypertension (p = 0.002) and diabetes (p = 0.002). They also found that older age was associated with a greater risk of developing ARDS and that death was increasingly likely due to a less rigorous immune response [3]. Another Wuhan retrospective cohort study by Zhou et al. reported that of 191 patients with COVID-19, 48% had other comorbidities, with hypertension the most common (30%), followed by diabetes (19%) and coronary heart disease (8%). They also found increased odds of death associated with older age (odds ratio 1.10, 95% CI 1.03–1.17; p = 0.004) [4].

Despite evidence of an association with other comorbidities, little is known about the impact of HIV infection on the clinical outcomes of patients infected with COVID-19. According to the Centres for Disease Control and Prevention (CDC), older populations and patients with compromised immune systems due to infections such as HIV are at a higher risk of contracting COVID‐19 [5]. Furthermore, the risk of complications due to SARS-CoV-2 infection is assumed to be even higher for HIV‐infected patients with low CD4 cell count and not on antiretroviral (ARV) regimens [5].

The first study to report an increased risk of mortality associated with HIV was a large cohort study in the UK that included data from more than 17 million adult patients in general practice records. It found that those with immunosuppressive conditions, including HIV, had a higher risk of death compared to those without (aHR 1.69; 95% CI (1.21–2.34) [6].

Another retrospective cohort study conducted in the UK by Bhaskaran et al. reported that although the crude risk of mortality from COVID-19 was the same among patients with HIV and non-HIV, after adjusting for age and sex, patients with HIV had a higher risk of COVID-19 related deaths (HR = 2·90, 95% CI 1·96–4·30; p<0·001) [7].

A systematic review and meta-analysis by Mellor et al. that looked at COVID-19 outcomes in patients living with HIV suggested a higher risk of mortality in HIV patients than non-HIV patients [HR = 1.95, 95% CI: 1.62–2.34]. The risk of mortality was also higher in a subgroup analysis of HIV patients from hospitalised cohorts [HR 1.60, 95% CI: 1.12–2.27]. However, data from the meta-analysis were insufficient on the effect of CD4 count and HIV viral load on COVID-19 outcomes [8]. Nevertheless, some cohort studies from Europe and North America have suggested that there is no increased risk of adverse outcomes in co-infected patients. Sigel et al. conducted a cohort study of all patients admitted with COVID-19 infection, comparing the outcome of HIV- positive versus HIV-negative patients in a large tertiary care hospital in New York. It was found that of 4402 patients admitted with laboratory-confirmed COVID-19, 88 were HIV-infected (2%), most of whom were black (40%) and Hispanic (30%). After adjusting for demographics and comorbidities, the authors concluded that HIV-infected patients did not have an increased risk of death than HIV-uninfected persons [9].

A retrospective study by Gervasoni et al. described the clinical characteristics and results of HIV-infected patients with a probable/proven diagnosis of SARS-CoV-2 infection who had been followed up in a referral hospital for the treatment of HIV infection in Italy [10]. Out of 6,000 HIV-positive patients in their database, 47 were diagnosed with probable or proven COVID-19 during the observation period. Of the 28 confirmed cases, 13 patients were hospitalised, 6 of them with severe illness, among whom there were only two deaths (15% of hospitalised). This outcome was compared to the crude mortality rate of 17% among HIV-negative patients admitted to the hospital [10]. Nonetheless, the interacting effects of HIV infection and COVID-19 in countries with a high burden of HIV are unknown.

Davies et al. reported a population cohort study using linked data from adults attending public health facilities in the Western Cape, South Africa [11]. The authors found that among 3.46 million public sector patients (16% HIV positive), 22,308 were diagnosed with COVID-19, of whom 625 died. Their analysis found that HIV was associated with an increased risk of COVID-19 mortality (aHR 2.14; 95% CI: 1.70, 2.70), with a similar risk across immunosuppression and viral load strata [11].

Except for the recent South African study, most studies reporting HIV and SARS-CoV-2 coinfection are few cohort studies from countries with low HIV burden. These studies are likely not relevant to sub-Saharan Africa, where most of the HIV population is younger, has a different pattern of comorbidities and has a different socioeconomic background. Although cohort studies from Europe and North America suggest that there is no increased risk of adverse outcomes, this does not reflect the results in countries with a high HIV burden, such as Kenya. With such significant knowledge gaps and an unknown trajectory of the COVID-19 pandemic, it becomes prudent to elucidate the role of HIV in understanding the COVID-19 pandemic, which requires an examination of specific cases at different scales.

## Materials and methods

This was a retrospective study conducted at the Aga Khan University Hospital, Nairobi (AKUHN), from March 2020 to December 2020. We included patients admitted to the facility with laboratory-confirmed COVID-19 infection using reverse transcriptase-polymerase chain reaction (RT-PCR) from a nasal pharyngeal swab specimen, sputum, or lower respiratory tract sample. Based on an institutional protocol for managing COVID-19 patients, HIV testing was to be carried out on all suspected or confirmed COVID-19 patients. Patients were verbally consented and documented for HIV-ELISA testing. The standard institutional method for HIV testing was HIV- ELISA 4^th^ Generation followed by a confirmatory 3^rd^ Generation. Approval for this study was obtained from the Institutional Scientific Ethics and Review Committee AKHUN (Ref:2020/IERC-113 (v2)) and since this was retrospective study an exemption was obtained and no consent was required.

The research enumerator extracted data from patient medical records and electronic health records. The data collected included demographics, vital clinical characteristics (temperature, heart rate, respiratory rate), symptoms (respiratory, cardiovascular, gastrointestinal, and central nervous system), comorbidities, and laboratory characteristics including CD4 and viral load. We used the CDC guidelines to define the clinical spectrum of COVID-19 infection, where asymptomatic or pre-symptomatic patients were defined as having positive RT-PCR but without symptoms consistent with COVID-19. Mild illness included patients with any signs and symptoms excluding shortness of breath or abnormal findings of chest radiography. However, patients with symptoms of difficulty in breathing, shortness of breath or abnormal chest radiography saturating ≥ 94% in room air, were classified as having moderate disease. Severe illness included those patients who were found to be desaturating <94% in room air with a ratio of arterial partial pressure of oxygen to the fraction of inspired oxygen <300mm Hg, tachypnoea >30/min or with >50% lung involvement in chest imaging. Individuals with respiratory failure, septic shock, or multiorgan dysfunction were categorised as critically ill.

## Results

A total of 582 patients were reviewed and included in the study. The mean age was 49.2 years (SD = 15.2). 68% of the patients were men. The prevalence of HIV was 3.7% (n = 22). The mean age of the HIV positive patients was 50.6 years (SD = 11.5), whereas for the HIV negative was 49.1 years (SD = 15.3). Most of the participants were of African race (87.3%) residing in Nairobi County (87.4%). Median CD4 [IQR] = 398.0 [94.0, 495.0] and viral load undetectable in 45.5% (10 patients), 20–35 copies in 31.8% (7 patients) and >10000 copies in 13.6% (3 patients). Table 1 describes the demographic characteristics of the patients.

**Table 1:** Demographic characteristics between the HIV positive and negative groups.

				HIV STATUS				P-Value
		Total (n = 582)		Positive (n = 22)		Negative (n = 560)		
***Age (years)***		48.9	[38.1, 59.3]	52.6	[45.4, 55.0]	48.6	[37.9,59.7]	0.570
***Age Categories (Years)***	***0–17***	11	1.9%	0	0.0%	11	2.0%	0.744
	***18–39***	156	26.8%	4	18.2%	152	27.1%	
	***40–69***	368	63.2%	17	77.3%	351	62.7%	
	***> 70***	47	8.1%	1	4.5%	46	8.2%	
***Gender***	**Male**	396	68.0%	16	72.7%	380	67.9%	0.816
	**Female**	186	32.0%	6	27.3%	180	32.1%	
***Race***	**African**	508	87.3%	22	100.0%	486	86.8%	0.095
	**Others**	74	8.1%	0	0.0%	74	13.2%	
***County (n = 573)***	**Nairobi**	501	87.4%	19	86.4%	482	87.5%	0.745
	**Others**	72	12.6%	3	13.6%	69	12.5%	

### Clinical characteristics

In terms of clinical characteristics (Table 2), the most common symptoms that appeared in the overall COVID-19 cohort were cough (58.1%), fever (45.2%), difficulty breathing (36.8%) and general body malaise (23.9%). The most common comorbidities were hypertension (28.5%), diabetes mellitus (26.1%), and heart disease (4.1%). Overall, most of the clinical characteristics did not show a significant difference between the HIV-positive vs HIV-negative groups, except for easy fatigability as a presenting symptom, which was more frequent in the HIV-positive group (13.6% vs 2.3%, p = 0.019).

**Table 2:** Clinical characteristics between the HIV-positive and negative groups.

				HIV STATUS				P-Value
		Total (n = 582)		Positive (n = 22)		Negative (n = 560)		
***General Symptoms***	Fever	263	45.2%	8	36.4%	255	45.5%	0.514
	Muscle Aches	32	5.5%	2	9.1%	30	5.4%	0.344
	General body malaise	139	23.9%	4	18.2%	135	24.1%	0.620
***Respiratory Symptoms***	Cough	338	58.1%	14	63.6%	324	57.9%	0.664
	Difficulty in Breathing	214	36.8%	4	18.2%	210	37.5%	0.073
	Runny Nose	6	1.0%	0	0.0%	6	1.1%	1.000
	Sore Throat	48	8.2%	1	4.5%	47	8.4%	1.000
	Chest Pain	95	16.3%	7	31.8%	88	15.7%	0.070
	Wheezing	6	1.0%	0	0.0%	6	1.1%	1.000
***Gastrointestinal Symptoms***	Vomiting	56	9.6%	4	18.2%	52	9.3%	0.152
	Diarrhoea	46	7.9%	3	13.6%	43	7.7%	0.406
	Abdominal Pain	26	4.5%	1	4.5%	25	4.5%	1.000
***Cardiovascular Symptoms***	Palpitations	15	2.6%	0	0.0%	15	2.7%	1.000
	Easy Fatigue	16	2.7%	3	13.6%	13	2.3%	0.019
***CNS Symptoms***	Headache	89	15.3%	4	18.2%	85	15.2%	0.761
	Confused	3	0.5%	0	0.0%	3	0.5%	1.000
**Respiratory Exam (n = 578)**	Chest clear	371	64.2%	12	2.1%	359	62.1%	0.569
	Crepitations	143	24.7%	7	1.2%	136	23.5%	
	Other abnormal findings*	64	11.1%	3	0.5%	61	10.6%	
**Vital Signs**	Temperature (n = 573)	36.7	[36.4, 37.2]	36.8	[36.5,37.7]	36.7	[36.4,37.2]	0.539
	SPO2 (n = 563)	94.0	[89.0, 96.0]	94.0	[87.0, 96.0]	94.0	[89.0, 96.0]	0.930
	Respiratory Rate (n = 572)	18.0	[18.0, 20.0]	19.0	[18.0, 20.0]	18.0	[18.0, 20.0]	0.474
	Heart Rate (n = 577)	94.0	[82.0, 106.0]	95.0	[79.0, 112.0]	94.0	[82.0, 106.0]	0.882
***Known Comorbidities***	Diabetes	152	26.1%	8	36.4%	144	25.7%	0.321
	Hypertension	166	28.5%	6	27.3%	160	28.6%	1.000
	Heart Disease	24	4.1%	0	0.0%	24	4.3%	1.000
	COPD	1	0.2%	1	4.5%	1	0.2%	1.000
	Renal Disease	19	3.3%	0	0.0%	19	3.4%	1.000
	Cancer	13	2.2%	1	4.5%	12	2.1%	0.397
	Rheumatology Disorder	3	0.5%	1	4.5%	2	0.4%	0.109
***Renal Disease***	Stage 2	1	5.3%	0	0.0%	1	0.2%	1.000
	Stage 3	5	26.3%	0	0.0%	5	0.9%	
	Stage 4	13	68.4%	0	0.0%	13	2.3%	

*Other abnormal findings include wheeze and bronchial breath sounds

The average length of stay for all participants was 6 days (IQR = [3, 10]), with the HIV-positive group taking 5 days (IQR = [3, 11]) and 6 days (IQR = [3, 10]) for the HIV-negative group (p = 0.833). Table 2 shows the clinical characteristics of the two groups. Based on the severity of COVID-19, 49.5% (228) were mild, 3.3% (19) moderate, 45.0% (262) severe and 2.2% (13) were critical. There was no statistical significance in severity between the HIV positive and negative groups (p = 0.558). Only 5% of the participants had died from the overall patient cohort. All deaths occurred in the HIV-negative group and none in the HIV-positive group (Age (Median [IQR]) = 68.5 [52.2, 82.3] years. Table 3 describes the length of stay, disease severity and mortality.

**Table 3:** Length of stay, disease severity and mortality.

				HIV STATUS				P-Value
		Total		Positive (n = 22)		Negative (n = 560)		
***Outcomes***	Recovered	553	95.0%	22	100.0%	531	94.8%	0.619
	Died	29	5.0%	0	0.0%	29	5.2%	
***Duration to Death (n = 29)***		6.00	[4.00, 10.00]	—	—	6.00	[4.00,10.00]	—
***Duration to Discharge (n = 553)***		6.00	[3.00, 10.00]	5.00	[3.00, 11.00]	6.00	[3.00, 10.00]	0.833
***Overall During Hospital Stay requirement***								
Mechanical Ventilation		38	6.5%	3	13.6%	35	6.3%	0.167
Ionotropic Support		7	1.2%	0	0.0%	7	1.3%	1.000
Oxygen		320	55.0%	10	45.5%	310	55.4%	0.389
***COVID-19 Severity***								
***Mild***		288	49.5%	10	45.5%	278	49.6%	0.558
***Moderate***		19	3.3%	0	0.0%	19	3.4%	
***Severe***		262	45.0%	11	50.0%	251	44.8%	
***Critical***		13	2.2%	1	4.5%	12	2.1%	

## Discussion

This study found a 3.7% prevalence of HIV in patients with COVID-19 who came to the Aga Khan University Hospital, Nairobi. The demographic characteristics of HIV-positive and HIV-negative COVID-19 patients were similar. There were no significant differences in length of hospital stay, disease severity and mortality.

The 3.7% prevalence of HIV in patients with COVID-19 who present at the AKUHN is less than the cumulative prevalence of HIV in Nairobi County, which according to the Kenyan HIV estimates report of 2018, was 6.1% [12]. This could suggest that HIV status is unlikely to represent a significant influencing factor in COVID-19 infection. Nevertheless, it is also important to consider that this finding could be the result of the sociodemographic characteristics of the patients who present at the hospital. Since AKUHN is a private, not-for-profit hospital, patients tend to have a relatively higher socioeconomic standing than the general population. A clear social gradient of disease has been demonstrated in the epidemiology of HIV infection. Patients with HIV coinfection did not differ from those without HIV, showing that if HIV is well treated, the results of COVID-19 infection would be similar to those of the general population.

The demographic characteristics in HIV-positive vs HIV-negative groups were similar in terms of age, gender, race and county of residence. Clinically, the most common presenting complaints were cough (58.1%), fever (45.2%), difficulty breathing (36.8%) and general body malaise (23.9%). Furthermore, most of these were similar between the study groups, with the only exception being easy fatigability as a presenting symptom that was more frequent in the HIV-positive group (13.6% vs 2.3%, p = 0.019). This finding is consistent with those of other studies, which suggest little or no difference in the clinical characteristics of COVID-19 in HIV-positive versus HIV-negative patients [10, 13–15].

The most common comorbidities were hypertension (28.5%), diabetes mellitus (26.1%), and heart disease (4.1%), which is consistent with previously published research on COVID-19 [3, 4, 16, 17]. It also likely reflects that these illnesses tend to be relatively common in the general population.

In terms of clinical outcomes, our finding of no significant difference is similar to that of other cohort studies [9, 10, 13–15, 18, 19]. However, it contradicts the findings of two extensive systematic reviews [12, 13]. One possible explanation for this is the likely higher socioeconomic status of the participants in our sample, as discussed above. Because of this, it is plausible that they were more likely to receive quality care, be virally suppressed and therefore immunologically competent, reducing the risks posed by infections such as COVID-19. Another noteworthy consideration that might explain our findings is that due to the relatively low prevalence of HIV, there was a sample size of only 22 participants in the HIV-positive group, limiting the statistical power to detect differences in clinical outcomes. This is compounded by the fact that the base rate of many of these clinical outcomes is relatively small. Other studies have found similar sample size limitations [10].

The main strength of this study is that to our knowledge, it is the first and only study of its kind conducted in the East African region and one of only two conducted in Africa.

Notwithstanding this, we also recognise some limitations. First, the small sample of HIV patients could have limited the statistical power of the study to detect significant differences, especially in relatively rare outcomes. Secondly, the use of the AKUHN as the only study site likely introduced some level of selection bias and may have limited the generalizability of the findings. Finally, no information was available regarding the immunologic status or ARV regimen in the HIV-positive participants, which limited the ability to conduct subgroup analysis. Future studies should work on addressing these limitations in order to move the knowledge on this topic forward.

## Conclusion

This study found a 3.7% prevalence of HIV in patients with COVID-19 who came to the Aga Khan University Hospital in Nairobi. HIV-positive and HIV-negative COVID-19 patients were similar demographically and in clinical characteristics. There were no significant differences in clinical outcomes. We wish to postulate that if HIV is well treated, the outcomes of COVID-19 infection would be similar to those of the general population.

Future studies should work towards achieving larger samples, include multiple study sites and conduct subgroup analyses based on the immunological status of HIV-positive patients.

## Supporting information

S1 Data (CSV)
